# New Appliances and Things Medical

**Published:** 1900-08-11

**Authors:** 


### NEW APPLIANCES AND THINGS MEDICAL.

[We shall be glad to receive, at our Office, 28 & 29, Southampton Street, Strand, London, W.C., from the manufacturers, specimens of all new preparations and appliances which may be brought out from time to time.]

### GLOBENA MEAT PREPARATIONS.

(Cooper and Co., 80, Gloucester Road, London, S.W.)

The Globena meat preparations include beef essence, chicken essence, meat lozenges, and meat juice. From a careful examination which we have conducted with respect to these invalid specialities, we are fully satisfied that they are of excellent quality and exceptional purity. The beef and chicken essence should be particularly useful for serious cases of illness, as, although concentrated, they are pleasant to the taste and easily swallowed and absorbed. The Globena meat juice is a very good sample of this popular form of nutriment, and it should be of great value in the treatment of such cases as gastric ulcer, in which the stomach is intolerant of solid forms of food, and yet requires a maximum of nutriment with a minimum of bulk. The meat lozenges constitute a useful variety of concentrated food, and are pleasant to the taste; travellers will appreciate their refreshing and sustaining qualities.

